# Baobab isotope records and rainfall forcing in Southwest Madagascar over the last 700 years

**DOI:** 10.1371/journal.pone.0331274

**Published:** 2026-03-10

**Authors:** Estelle Razanatsoa, Lindsey Gillson, Grant Hall, Malika Virah-Sawmy, Stephan Woodborne

**Affiliations:** 1 Plant Conservation Unit, Department of Biological Sciences, University of Cape Town, Rondebosch, South Africa; 2 Leverhulme Centre for Anthropocene Biodiversity, University of York, United Kingdom; 3 Stable Isotope Laboratory, Mammal Research Institute (MRI), University of Pretoria, South Africa; 4 Humboldt-Universitat zu Berlin, Geography, Germany; 5 iThemba LABS, Johannesburg, South Africa; Woods Hole Oceanographic Institution, UNITED STATES OF AMERICA

## Abstract

Highly resolved climate records for Madagascar are scarce but are essential for understanding of rainfall drivers over time and assessing the risks and likely trajectories of future climate change. We measured variation in the carbon isotopes of baobabs (*Adansonia* spp.) which reflect rainfall in southwest Madagascar. The record indicates a decreasing trend of rainfall over the last 700 years with high variability at a centennial-scale. The duration of wetter periods decreased over time with the wettest periods between 1350–1450 CE, after the onset of the Little Ice Age, while the driest period occurred between 1600–1750 CE, during the Maunder Minimum. The results suggest that decadal to centennial rainfall variability in southwest Madagascar is dominated by tropical forcing rather than subtropical forcing. Wetter periods are regulated by the movement and migration of easterly winds linked to the Intertropical Convergence Zone, while dry periods are influenced by the effect of the Pacific Decadal Oscillation linked to the El Niño Southern Oscillation and the sea surface temperature variation in the Southwestern Indian Ocean. The Southern Annular Mode is significantly correlated with the record, but its effect was only visible at the beginning of the record around 1300 CE. This evidence provides a new understanding of rainfall across southern Africa and the interaction of global forcing with regional factors. Further investigation is required to improve tree chronology from Southern Hemisphere and understand the migration of the westerlies and its potential future effect on the rainfall in Madagascar. Understanding the interplay between tropical and other rainfall forcings will be essential in assessing likely scenarios of resilience, and adaptive capacity of social-ecological systems in Madagascar.

## 1. Introduction

**Fig 1:**
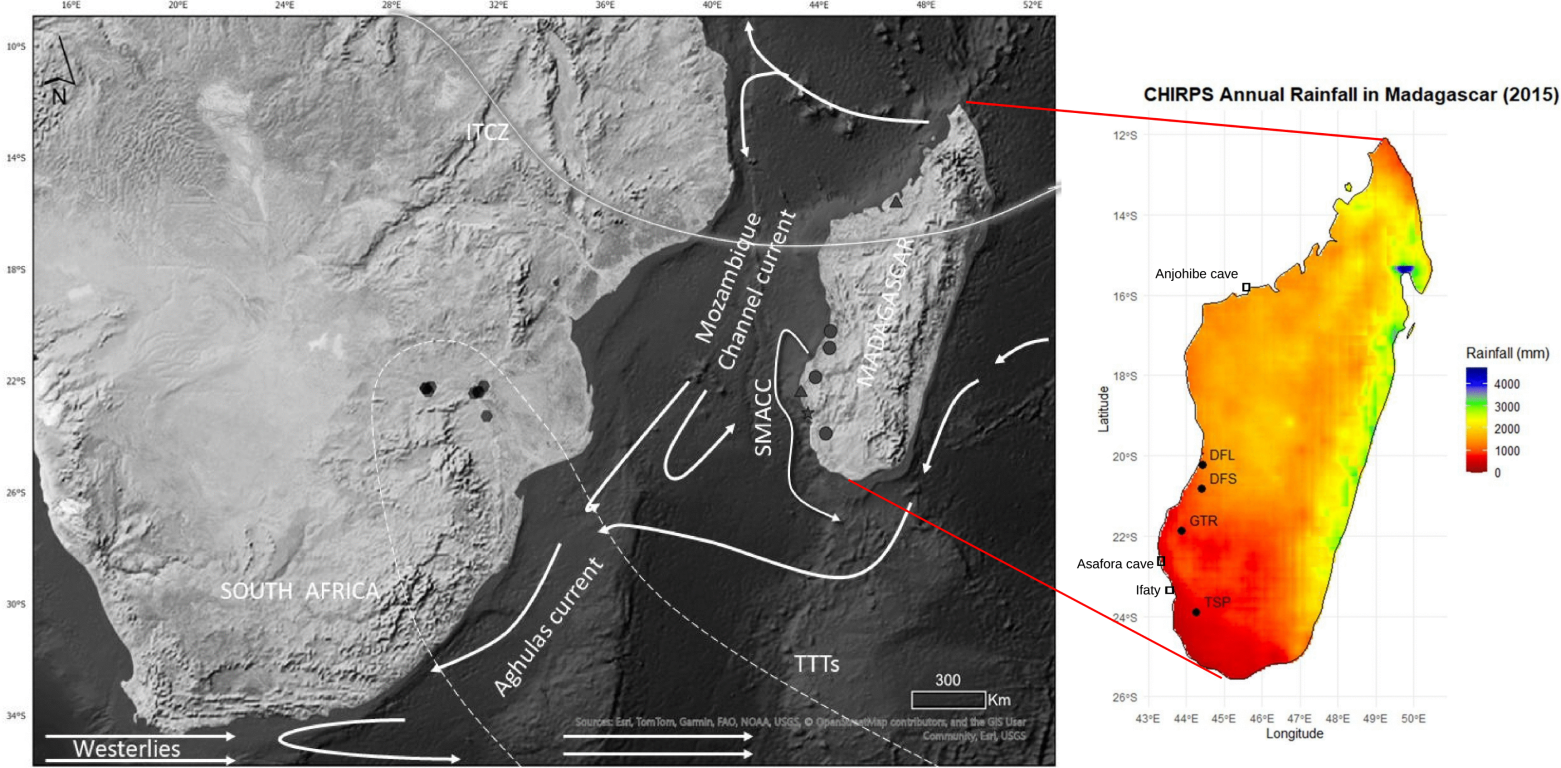
Sampling sites and austral summer synoptic features in Southern Africa. (A) Austral summer synoptic features in southern Africa, including the Inter Tropical Convergence Zone (ITCZ), Tropical Temperate Troughs, Southwest Madagascar Coastal Current (SMACC), Agulhas Current and Mozambique Channel Current. along with the published tree records from southern Africa (hexagons). The data for the basemap is downloaded from Natural Earth I with Shaded Relief and Water (https://www.naturalearthdata.com/downloads/50m-raster-data/50m-natural-earth-1/) and is similar but not identical to the original image and is therefore for illustrative purposes only **(B)** Madagascar rainfall gradient based on CHIRPS Datasets for 2015 [13] showing the four trees investigated in this study (circles) and the location of other proxy records used in this study (rectangles).

The African continent has been tangibly affected by climate change in the last decades, manifested by increasing temperatures and rainfall variability [1–4]. In parts of southern Africa, decreased rainfall with more pronounced and recurrent severe drought events are expected in the near future, e.g., [1,5–9]. Understanding rainfall drivers requires knowledge of local and regional synoptic changes that occur at decadal to centennial scales [10]. Madagascar plays a role in the regulation of climate across southern Africa through its topographic influence on the Mozambique Channel Trough (MCT) [11]. Madagascar has an east-west rainfall gradient associated with its topography which reduces the influence of easterly trade winds that bring moisture from the equatorial Indian Ocean [12], Fig 1. A north-south rainfall gradient derives from the north-westerly monsoon as the ITCZ crosses Madagascar to 20°S during the austral summer [10,14–16]. While the southwest region derives moisture from the monsoon and tropical cyclone events in the South Indian Ocean (east Madagascar), and the Mozambique Channel (west-southwestern Madagascar) [17], it is the driest area on the island with <600 mm of rainfall per year with recurrent drought events [16,18]. The southern region of Madagascar has experienced limited rainfall at least in the last few decades [9,19–21]. The region is also vulnerable to future global climate changes as simulations suggest more severe and longer dry seasons in the tropics, and an average global rise in temperature of 1.5*°C estimated to be reached* by 2030 [1,22,23]. This forecast is associated with large uncertainty due to a lack of understanding of seasonal, annual, decadal, and centennial rainfall trends, and this prevents proper assessment of adaptation and hazard reduction in the area [9,19-21]. The region is also vulnerable to future global climate changes as simulations suggest more severe and longer dry seasons in the tropics, and an average global rise in temperature of 1.5*°C estimated to be reached* by 2030 [1,22,23]. This forecast is associated with large uncertainty due to a lack of understanding of seasonal, annual, decadal, and centennial rainfall trends, and this prevents proper assessment of adaptation and hazard reduction in the area [9].

On an annual to decadal scale, droughts in southern Africa have been associated with El Niño Southern Oscillation (ENSO, [8]). Austral summer rainfall is controlled primarily by the seasonal interplay between subtropical high-pressure systems and the migration of easterly flows associated with the Intertropical Convergence Zone (ITCZ) [24,25]. The position and intensity of tropical-temperate troughs (TTTs) and their associated cloud bands that form in response to this interplay [26] are closely linked to sea surface temperature (SST) conditions in the Southwest Indian Ocean (SWIO) [27–29]. These synoptic dynamics are derived from observational data. Longer term data are needed to understand trends and the interplay between the spatial dynamics of rainfall and the different drivers at decadal and centennial scales. This is especially true for Madagascar where rainfall variability is intrinsically high; there is a high dependence in rain-fed agriculture; and environmental vulnerability is high.

The limited climate analyses from the island show drying trends at millennial, centennial to decadal scales [16,18,30] ENSO and the seasonality of the ITCZ are essentially tropical forcing mechanisms, but recent evidence suggests that under glacial climate forcing, there is an influence from the sub-tropics that is related to the latitudinal position of the southern hemisphere westerlies [31]. The South Annular Mode (SAM) is a position index of the westerly vortex, but little is known about its past and current effect on rainfall in the region [28] although it is known to affect circulation on weekly to centennial time scales (e.g., [32,33].

### Paleoclimate proxy records

Paleoclimate records contain information at various temporal and spatial scales, which enables the interpretation of trends, variability, and the underlying processes within the climate system. Paleoclimate proxy records derived from ice cores, e.g.,[34,35], lake and wetland sediments, e.g., [36,37], and speleothems, e.g., [10,38,39] provide a deep time record of global-scale climate forcing, while coral reefs, e.g., [40] and tree rings, e.g., [41,42] yield shorter, more localised records of climatic responses. In temperate environments, abundant records of tree ring widths and varved sediments growth can be used to infer rainfall regimes, but records with similar resolution are rare in tropical ecosystems. Recently the possibility of reconstructing past climate variability using carbon isotope ratios (*δ*^13^C) from the growth rings of subtropical trees has emerged [43–45]. This increases the potential for paleohydrological reconstruction in the subtropics, and the approach holds the potential to address the climate data deficit in Madagascar which has numerous species of long-lived, dry adapted trees such as the baobab [46–48].

### Carbon isotope in tree rings as a climate proxy

The use of *δ*^13^C as a climate proxy in tree growth is based on environmental regulation of carbon isotope fractionation during photosynthesis. Fractionation is controlled by stomatal conductance, which manifests in the ratio of leaf internal CO_2_ concentration and the atmospheric CO_2_ concentration (*c*_*i*_/*c*_*a*_). Many environmental factors control stomatal conductance [49,50], but in ecosystems where water stress is the main control, reducing the *c*_*i*_/*c*_*a*_ increases the *δ*^13^C values of plants (more positive) in dry conditions. In low rainfall regions, water stress responses in trees relate to edaphic water availability which is directly linked to rainfall [49]. In the southern African subtropics, the *δ*^13^C value of a specific growth ring in a tree potentially reflects the rainfall conditions during the period when the ring was formed. A negative correlation consistent with the theoretical expectation was found between rainfall and the *δ*^13^C values of baobab rings in Southern Africa [44]. Low *δ*^13^C values infer higher rainfall while high *δ*^13^C values are indicative lower precipitation, but a direct transfer function accounting for evaporation, rechange and runoff has not been established, and so the proxy record reflects relative changes in effective rainfall through time. The isotopic records from southern African baobabs allow the investigation of the local rainfall drivers of rainfall through multiple records, and they reveal decadal, multi-decadal and centennial variability of rainfall over the last 1000 years [44,45]. In this paper, we describe new proxy rainfall records for the southwest region of Madagascar during the last millennium based on *δ*^13^C time series from several baobab trees and evaluate the potential drivers of centennial variability of rainfall in this area.

## 2. Material and methods

### 2.1. Study setting

This research focuses on southwest Madagascar, the driest region of the island (Fig 1) with permission from the Ministry of Environment and Sustainable Development and the Directions des aires protegees terrestres in Madagascar for field research under Permit number 211/15/MEEMF/SG/DGF/DAPT/SCBT. The area has a semi-arid climate with irregular rainfall [12,14] decreasing from 600 mm to less than 300 mm per year towards the south and the coast. Most of the rainfall occurs during the austral summer (November – March) with monthly rainfall between 300 mm for January and <2 mm in June, July, and August with an above average dry season rainfall recorded between 1983 and 1990, while wet seasons had elevated precipitation 2000–2015 [51]. In seasonally dry climates, a maximum in biomass is to be expected for a wet season of optimal length, for which the limitations imposed by both water availability and growth duration are at a minimum [52]. The implication is that radial growth rates will vary from season to season depending on rainfall amount and duration, but also over longer time periods where successive wet or dry years will accelerate or slow growth at a scale that cannot be resolved in the absence of annual growth rings. Nevertheless, the variation of isotope δ^13^C in a tropical context has been associated with rainfall variability [53].

This region supports three baobab species, *Adansonia grandidieri, A. za* and*, A. rubrostipa,* and all three species offer the opportunity to establish paleoclimate records as they are long-lived with distinct radial growth rings [44,45,54].

### 2.2. Sample collection

Sampling was conducted in 2015 on four living trees following a north-south transect of southwest Madagascar to cover the regional climate. The trees were coded as DFL, DFS, GTR and TSP based on their location from north within the dry forest vegetation to the spiny thicket vegetation in the south (shown in Fig 1; Table 1). The trees were selected and cored, based on their large size (>7 m in circumference) and location (>1 km from any possible surface water source, such as marshes and lakes that could obscure the rainfall signal due to the buffering effects of local hydrology). In addition to official permits for sample collection and exportation from the Ministry of Environment and Sustainable Development of Madagascar and Madagascar National Parks, local communities were consulted and gave permission for the sampling to proceed. Cores were carefully extracted from selected trees using a Haglöf 10 mm diameter increment borer at a position of 1.30 m above the ground. After core extraction from each tree, the hole was sealed with a commercial tree sealant to prevent any potential damage to the tree by insects or fungus infestation [55].

**Table 1:** Core information from trees sampled along a north-south transect in southwestern Madagascar.

Species and sample ID	Coordinates	Elevation (m)	core length (cm)	Tree circumference at breast height (m)	Number of δ^13^C samples	Number of ^14^C AMS dates
*A.gran* 01 – DFL	−20.2224167 ºS 44.4277500 ºE	25	97.5	8.8	433	12
*A.gran* 04 – DFS	−20.8207222 ºS 44.3940000 ºE	20	92	11.9	445	12
*A.gran* 05 – GTR	−21.8602778 ºS 43.8670556 ºE	23	91	12.2	450	13
*A. za* – TSP	−23.8863889 ºS 44.2583889 ºE	88	139	7.7	706	15

### 2.3. Tree chronology

The chronologies of the cores were determined from 52 AMS radiocarbon dates: respectively 12 for DFL, 12 for DFS, 13 for GTR and 15 for TSP (Table in S1 Table). Samples for radiocarbon dating were selected based on conspicuous shifts in the δ^13^C time series. Samples were processed at iThemba LABS in Johannesburg (South Africa). Samples were pre-treated using the acid-base-acid (ABA) method [56]. This treatment consists of the use of acid 0.5M HCl at 60°C, followed by a weak base of 0.1M NaOH at 60°C, to dissolve humic acids and finally a hot acid wash of 0.5M HCl at 60°C, removing any carbonates that precipitated from modern atmospheric carbon dioxide. Following each step, the samples were rinsed until a neutral pH was obtained [56]. The dates were calibrated with the 2020 Southern Hemisphere calibration from the online calibration programs CALIB 14C version 7.10 [57] and the Calibomb program (http://calib.org/CALIBomb/) from Queens University, Belfast, which uses the bomb carbon dataset of [58].

The age model uses wiggle matching between the trees to constrain possible radiocarbon calibration intercepts, as was used in previously published tree records from southern Africa [44,45] and the detailed approach to the age-depth modelling is described in [59]. Linear age interpolation of the chronology was used to assign an age to each δ^13^C analysis [59]. The approach is a pragmatic attempt to use the climate data as additional *a priori* constraints on the age of samples. In Bayesian age models, the relative chronology (*x* is known to be older than *y* based on stratigraphy, or in the case of trees, the radial growth) is used as an *a priori* constraint, and these methods provide a means of propagating analytical errors in a series of radiocarbon dates. Our approach notes that within windows of 50–100 years (the kind of error that may be expected in a radiocarbon age model) there are sequences of extreme rainfall and drought events that are common across different trees, as is the atmospheric *δ*^13^C Suess Effect, and these should be presumed to be synchronous because they are driven by large scale synoptic or earth system dynamics (Table 2). In this way climatology in addition to the radiocarbon dates provide many more *a priori* constraints on the chronology than the traditional Bayesian models based solely on the radiocarbon dates. None of the existing Bayesian age model packages provide a convenient way to integrate these additional constraints. Bayesian age models produced monotonic growth rate changes that did not integrate the ecological and physiological growth of the trees, and led to patent discrepancies in the age assignations for common signals across the trees. The argument has a risk of circularity because the chronology is required to generate the climate record, and the climate record is required to refine the chronology, but all attempts to reconcile climate sequences are constrained by the requirement that the age model must fall within the 1-sigma calibration ranges for the radiocarbon dates (with the exception of patent erroneous radiocarbon dates that show clear age inversions in the radial growth of trees, and which should be dismissed as errors, possibly in sample labeling). An implication of this approach is that a date on an individual tree becomes a constraint on the age model of all the trees for that particular time period. The major flaw in the approach is the inability to calculate errors on the age model, although the additional constraints from the climate record, and the demand for coherence in large scale climate events across trees likely yields a tighter chronology than a traditional Bayesian model based only on the within-tree radiocarbon analyses alone. The record obtained from the isotopic values and the age model, was therefore subject to a 21-year-biweight mean analysis which suppresses uncertainties in the age model and also the localised effect of synoptic changes over stochastic weather processes [44,45]. The approach prevents any analysis of temporal patterns that are resolved at less than decadal time scales.

**Table 2:** Chronological tie points of the 4 trees. GTR ring 380 has been assigned two ages which represents a growth hiatus as per [60]. See the illustrated age model in Fig 2.

Species and core ID	Year	Sample number
*A.gran* 01 – DFL	2005	10
	1625	265
	1280	450
*A.gran* 04 – DFS	2015	1
	1890	130
	1590	380
	1210	450
*A.gran* 05 – GTR	2015	18
	1750	380
	1500	380
	1340	450
*A. za* – TSP	2015	1
	1890	280
	1620	600
	1300	706

### 2.4. Stable carbon isotope analysis

The *δ*^13^C measurements were conducted at the Stable Isotope Laboratory at the University of Pretoria. Each core was mounted on a wood backing panel so that their axial orientation of the baobab core could be seen. The exposed half of the core was sub-sampled from the bark to the pith while being sensitive to the observable direction of the tree growth ring. The remaining half of the core are preserved as an archive. The number of samples per core is provided in Table 1. Each sample was placed in an individually labelled reaction vessel and subject to a Soxhlet extraction in a 2:1 toluene/ethanol mixture to eliminate the mobile and soluble components in the wood. This was followed by α-cellulose extraction in 17% sodium hydroxide (NaOH) and 10% sodium chlorite (NaClO_2_) at different concentrations to remove the lignins and eliminate the hemicellulose of the wood. The last steps consisted of covering the samples with 1% hydrochloric acid (HCl) solution for 10 minutes, rinsed and dried overnight at 70 °C [61,62]. Once dried, aliquots of the α-cellulose were weighed (0.050–0.060 mg) using a Mettler Toledo MK5 microbalance and folded into tin capsules before isotopic analysis. The samples were combusted at 1020°C in an elemental analyzer (Flash EA 1110 Series) coupled to a Delta V Plus stable light isotope ratio mass spectrometer, via a ConFlo III system (all equipment supplied by Thermo Fischer, Bremen, Germany). An in-house running standard of Malaysian wood (*Shorea superba*) with an isotopic value of −28.4‰ was used [44,45,62,63] to calibrate the results. A blank and in-house standard sample which was calibrated against a large number of international standards was run after every 12 samples. The results were reported in *δ*^13^C relative to Vienna-PDB (VPDB) and expressed as per mille according to the formula:

*δ*^13^C = (R_sample_/ R_standard_ -1)٭1000

Where *δ*^13^C is the isotopic composition of the sample and R indicates the ratio of ^13^C/^12^C in the sample. Sample replication including sample pre-treatment and error in the analysis were <0.2‰. This is based on a total of 1936 analyses conducted for the overall project, giving 161 standards approximately. The precision achieved here is similar to that reported by other stables light isotope laboratories.

### 2.5. Isotope correction and analysis

Tree physiological responses to environmental conditions are reflected in the *δ*^13^C ratios of the wood tissue [61], but the carbon isotope ratios are also affected by changes in the isotopic ratio of atmospheric CO_2_ over time [49] and variation in intrinsic water-use efficiency (iWUE) in response to elevated atmospheric CO_2_ concentrations since the industrial revolution [64,65]. The isotopic data derived from each tree therefore needs to be corrected to isolate the local environmental response. This correction compensates for the *δ*^13^C_atm_ variations using the global record of Belmecheri & Lavergne [66] and is a simple normalisation to pre-industrial *δ*^13^C_atm_ values [61]. In this study this is assigned to 1748 (the inflection date between pre- and post-industrial atmospheric changes in the Belmecheri & Lavergne dataset [66]). In addition to the measured change in *δ*^13^C_atm_, there is also an increase in the atmospheric concentration of CO_2_ (*c*_a_), which affects the intercellular CO_2_ concentration (*c*_i_). Since the rate of carbon assimilation, and accordingly the *δ*^13^C ratio of wood tissue from each growth ring, is linked to the *c*_*i*_*/c*_*a*_ ratio [61], reduced water transpiration is associated with elevated *c*_*a*_ during photosynthesis, which increases the iWUE of the plant. The correction used in this study followed the method established by Woodborne and colleagues [45], which is equivalent to the *δ*^13^C pin (“preindustrial”) correction method [67].

A 21-year biweight mean (a weighted mean that considers the *δ*^13^C values from the decade prior to, and after the date in question) was calculated to emphasize the decadal and multi-decadal pattern in the record. The biweight mean also accommodates the possible age model errors that arise from using a linear growth model to approximate a growth pattern that is likely punctuated by slight variations in growth rates.

The isotopic time series provides a regional decadal record of effective rainfall for the four trees. Each tree experienced unique local climate conditions over the last 700 years; and the composite record allows evaluation of the common and wider climate forcing by emphasizing commonalities in the low frequency component of the record. Change point analysis, a tool that have been proven to allow in detecting abrupt climate variations using the package change point in R was conducted on the composite records [68]. Following this, the composite record was compared with published data for a range of potential rainfall drivers including the local Southwestern Indian Ocean (SWIO) SST, and also equatorial climate shifts caused by the Indian Ocean Dipole (IOD) (Table 3).

**Table 3:** Paleoclimate forcing used for comparison with the baobab isotope records.

Data	Proxy	Time covered (CE)	Record length (years)	References
Sea Surface Temperature (SST, Ifaty)	Oxygen isotopes of coral	1660-1994	334	[69]
Southern Annual Mode Index (SAM)	Mid-latitude to polar domain proxy records	1000-2007	1007	[70]
Pacific Decadal Oscillation (PDO)	Tree rings	993-1996	1003	[71]
HadISST1.1 SST dataset (Index referring to IOD)	Calculated anomalous SST gradient between the western equatorial Indian Ocean and the south-eastern equatorial Indian Ocean. Based on coral isotopes and Ca/Mg ratios.	1981-2010	29	[72]

The identification of monotonic trends within the record was conducted using a non-parametric Mann-Kendall trend test [73,74] combined with a Least Squares Regression to evaluate the rate of change in rainfall per year in the regional records. In addition, gridded datasets downloaded from GPCC monthly total precipitation from around Betioky at 2.5° x 2.5° resolution were compared to the composite record. The correlation between the 21- year biweight mean and the 10-year moving average of a 30-year instrumental record (1970–2010) from Betioky meteorological station was calculated to validate the baobab records as a rainfall proxy for the southwest Madagascar. This difference in the smoothing method was conducted to accommodate the error in the chronology of the trees, as well as taking into consideration the short instrumental records. For correlations, significance at a level of 0.05 were accepted.

### 2.6. Inclusivity in global research

This research obtained a permission from the Ministry of Environment and Sustainable Development and the Directions des aires protegees terrestres in Madagascar obtained on the 27 August 2015 in collaboration with park managers and the University of Antananarivo (ESSA-FORET). Such permission is guaranteeing access to the trees, allowing sampling along with their exportation for analysis at the University of Cape Town. Additional information regarding the ethical, cultural, and scientific considerations specific to inclusivity in global research is included in the Supporting Information (S2 Checklist)

## 3. Results

**Fig 2:**
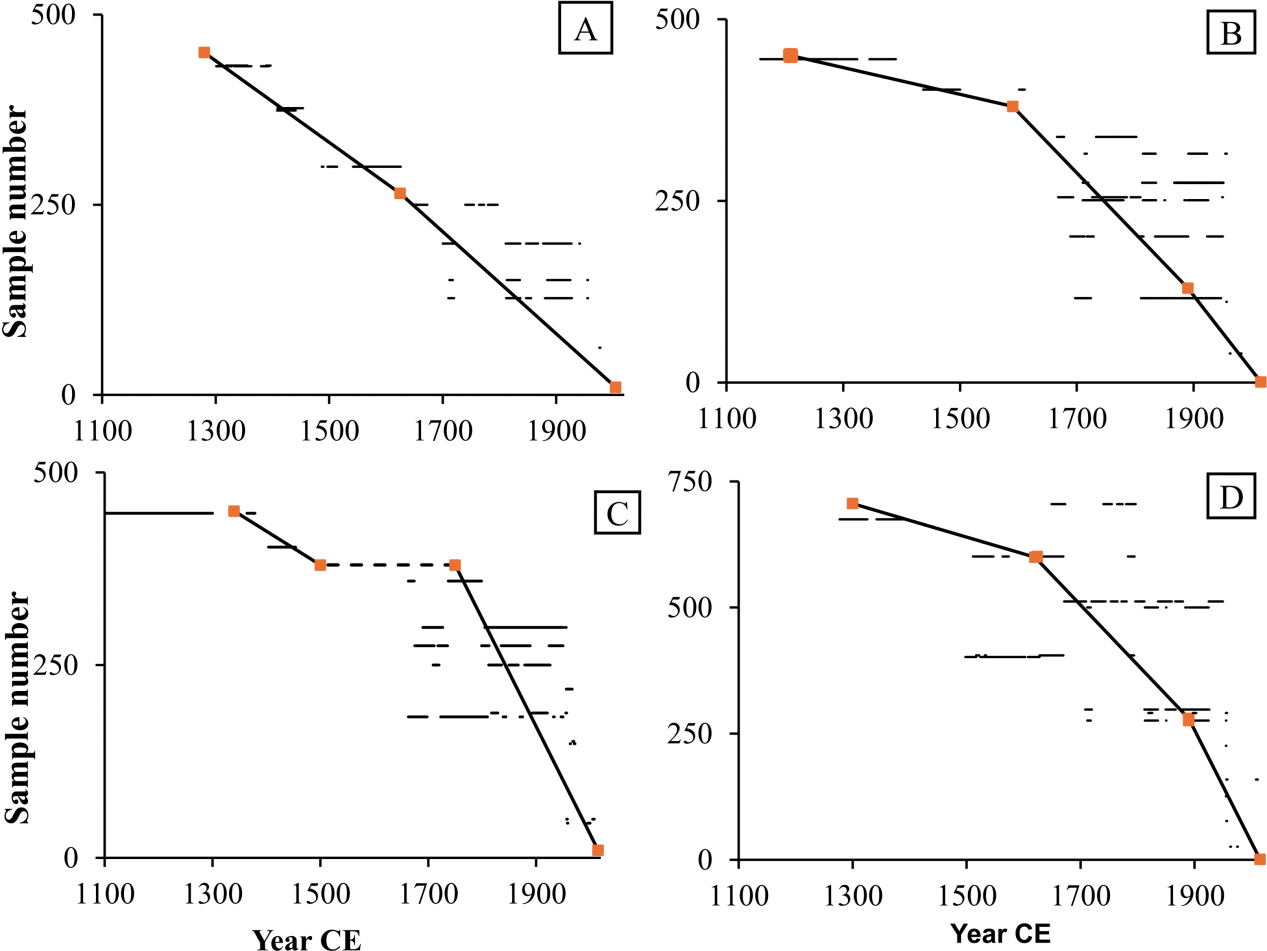
Age-models for (A) DFL, (B) DFS, (C) GTR with the hiatus indicated in dashed line, and (D) TSP, based on 52 radiocarbon dates. The horizontal lines are the 1-sigma calibration intervals for the radiocarbon dates. The bold line represents the age model that best intercepts the 1-sigma calibration range for the radiocarbon dates and align with the assumed chronological tie points (in orange squares) based on shared signals in the *δ*^13^C records.

The calibrated radiocarbon dates for the four trees suggest that they grew over the last 700 years (1302–2013 CE) (Table SI1). The most parsimonious age models, that reconcile the AMS dates and stable carbon isotope records, are shown in Fig 2. All the trees show linear growth over time except for GTR, which demonstrated a hiatus from 1500 CE to 1700 CE. This is not uncommon in baobabs [60].

**Fig 3:**
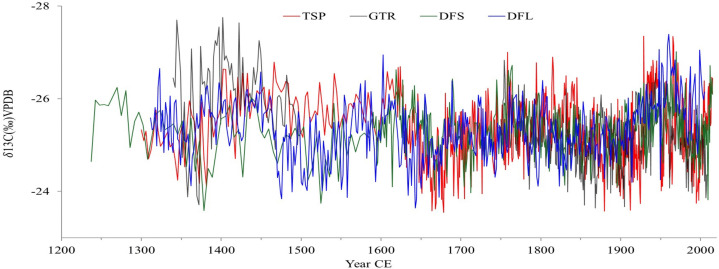
Corrected *δ*^13^C time series of four baobabs from southwest Madagascar with inverted y-axis indicating drier (less negative isotope value) and wetter conditions (more negative isotope value).

**Fig 4:**
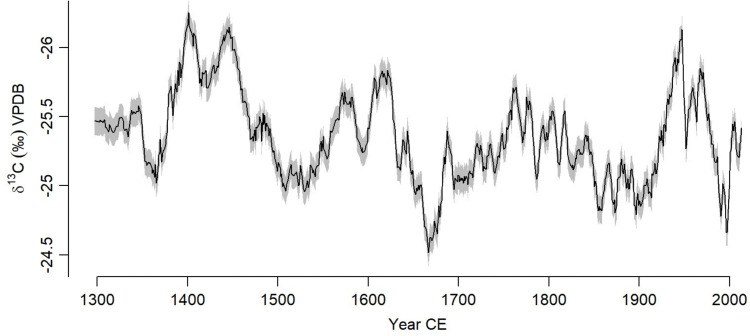
The composite record from four trees (black) with wetter periods (blue) and drier periods (yellow). Error bars represent standard errors.

The corrected *δ*^13^C time series from the four trees range between −26.2‰ (1400 CE) and −24.5‰ (1650 CE) with a mean of −25.3‰, and a variation of about 1.7‰ (Fig 3). The trend analysis on the composite *δ*^13^C records using the Mann-Kendall test shows that there is a marginal decreasing trend of the isotope data over time (S = −5.07, p < 0.01) with a difference of about −0.7‰ between 1300 CE and 2013 CE. A least square linear regression is significant (F = 80.6, p = 0.001) but with an R^2^ of 0.10. The change point analysis revealed a number of major shifts in the mean values of the composite isotope data becoming either more positive or negative in the time series (significant at 95%). These occur at approximately 980 CE, 1400 CE, 1480 CE, 1500 CE, 1630 CE, 1660 CE, and 1820 CE (Fig 4).

**Fig 5:**
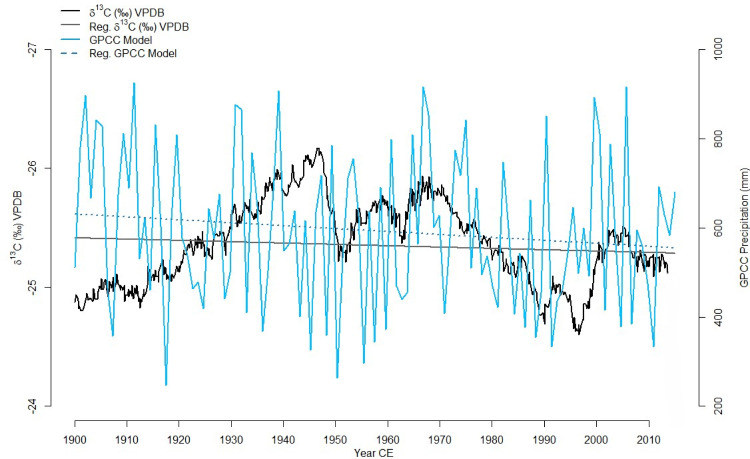
Comparison of the baobab*δ*^13^C records with existing model datasets: Black indicates the baobab *δ*^13^C composite records from 1900-2015 with linear regression indicated in grey. Blue and dark blue shows GPCC total precipitation at 2.5° x 2.5° resolution from around Betioky between 1900-2013 along with the associated linear model.

**Fig 6:**
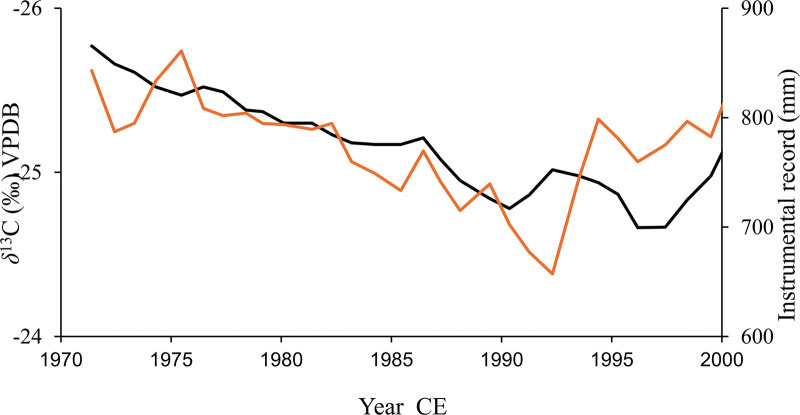
Validation of the baobab *δ*^13^C records as a rainfall proxy: Blue indicates the baobab *δ*^13^C composite records and red shows the 10-year moving average of 30 years (1970–2000) annual measurement of rainfall from the region.

The isotope biweight mean composite and the GPCC monthly total precipitation (Fig 5) along with the instrumental rainfall records (Fig 6) shows similar trends over time. A linear regression of precipitation data from the GPCC monthly total precipitation from 1900–present revealed a decreasing but not significant trend over time (β = −0.67, p = 0.17), with year explaining only ~1.7% of the variance (R² = 0.017). Conversely, the isotope data showed a small but significant increasing trend (β = +0.00112, p = 0.015) reflecting decreasing rainfall, though the model explains less than 1% of variance (R² = 0.0099). These results suggest subtle but differing temporal behaviours in the two proxies potentially associated with the associated ages. The *δ*^13^C records and the GPCC precipitation have shown similar trends in rainfall at decadal scale over the last 100 years particularly during the 1960–1980 CE despite the associated uncertainties in chronologies and the spatial resolution respectively. a slight decrease in rainfall as reflected by the precipitation value in both GPCC and instrumental records and the more positive isotope value were recorded around 1950 and then from 1970–1990 while more wetter periods are recorded around 1960 and 2000 (Fig 5, Fig 6). Such similar trends confirms that the data meets the theoretical expectation, and reaffirms the results obtained for baobab isotope controls on the African mainland [44,45], Fig 5, Fig 6.

The correlation analysis of potential rainfall drivers in Southwest Madagascar has been calculated (Table 4), while acknowledging that the precision of age model affects this calculation and only decadal comparisons are valid. There is a negative correlation between SST and the isotope data an overall significant positive correlation between SAM and the isotope records with recorded weak correlation since the 16^th^ century. Related to the Pacific Decadal Oscillation (PDO) reconstruction, no significant correlation has been recorded during the entire period but during the LIA negative PDO anomalies are associated with decreases in rainfall particularly between 1600–1750 CE (r = −0.30, p < 0.001), whereas in the record before and after this period, they are associated with increases in rainfall (r = 0.18, p = 0.004 and r = 0.18, p = 0.001 respectively) (Fig 8C).

**Table 4:** Correlation between baobab isotope data and various environmental indices. Pearson correlation coefficients (r) and associated p-values are shown. Sample size (n) indicates the number of data points compared for each dataset.

Dataset Compared with Baobab Isotope Data	Time Period (CE)	Sample Size (n)	Pearson Correlation (r)	p-value
Sea Surface Temperature (SST, Ifaty)	1660–1994	334	–0.22*	< 0.001*
Southern Annual Mode Index (SAM)	1000–2007	1007	0.16*	< 0.001*
Pacific Decadal Oscillation (PDO)	993–1996	1003	0.01	> 0.05
HadISST1.1 SST dataset (IOD Index)	1981–2010	29	–0.06	> 0.05

Note: * indicates significance at α = 0.05.*

**Fig 7:**
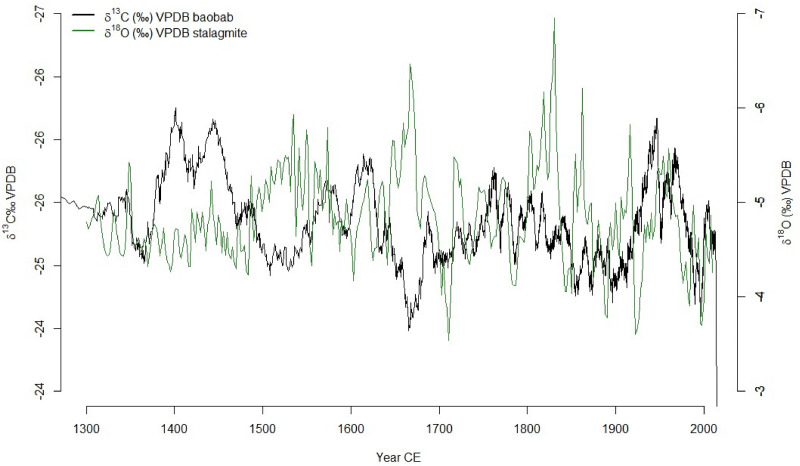
Comparison between baobab*δ*^13^C records from tree rings in southwest Madagascar (black) with stalagmite *δ*^18^O records from Asafora cave from the southwest coast and just southeast of the Velondriake Marine Reserve (green, from [75]).

**Fig 8:**
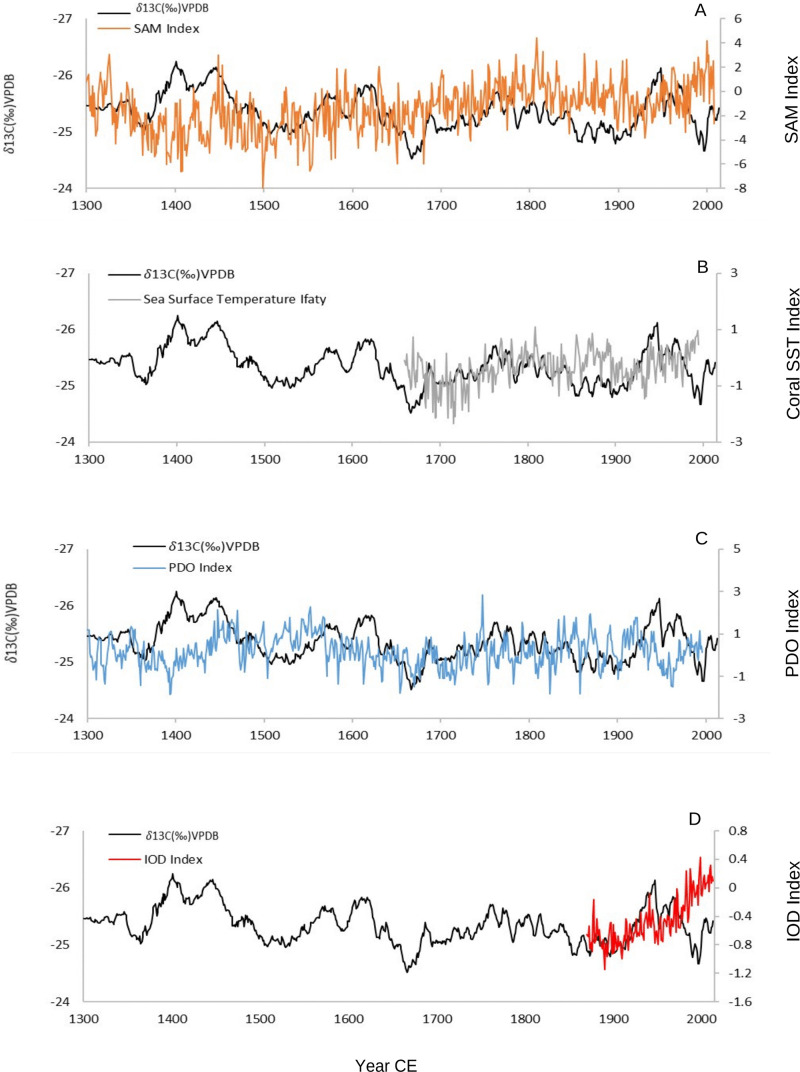
Comparison of the baobab *δ*^13^C records (black) with (A) the Southern Annual Mode (SAM) index (orange) [70], (B) the Pacific Decadal Oscillation (PDO) index (blue) [71], (C) Sea Surface Temperature (SST) from Ifaty, southwest Madagascar (grey) [69], and (D) HadISST1.1 SST dataset that refers to Indian Ocean Dipole variability (red, [72]).

## 4. Discussion

### 4.1. Rainfall record of southwest Madagascar for the last 700 years

The *δ*^13^C records and the GPCC precipitation shows similar trends in rainfall at decadal scale over the last 100 years particularly during the 1960–1980 CE despite the associated uncertainties in chronologies and spatial resolution respectively. Such similar trends confirm that the data meets the theoretical expectation and reaffirm the results obtained for baobab isotope controls on the African mainland [44,45], Fig 5. The baobab *δ*^13^C record can thus be interpreted as a proxy for local effective rainfall in southwest Madagascar, reflecting decadal to centennial variability in the last 700 years. Accordingly, lower *δ*^13^C values indicate wetter periods while more positive isotope ratios correlate to drier conditions.

The chronology of the four baobabs ranges from 1300 CE until 2013 with a hiatus in the GTR core from 1500 CE to 1700 CE. Punctuated growth models have been noted in other baobabs [60] and may arise because of attenuated growth rates over time, or possibly due to lobate development of the stem resulting in the reallocation of resources to another part of the tree. The GTR baobab was collected near the River Mangoky in southwest Madagascar where a sediment core was taken from nearby Lake Tsizavatsy. The age-depth model of the Tsizavatsy core shows a hiatus from 1400 to 1900, suggested to be associated with a regional drought event [76]. Although the other trees show positive isotope excursions (dry events) during this period, there are oscillations of wetter conditions covered by the present records (Fig 3). Notwithstanding the gap, the record from GTR prior to and after the hiatus was combined with the composite record of all trees to provide a full rainfall proxy record for the last 700 years for southwest Madagascar covering the Little Ice Age (LIA) [77,78] and the Anthropocene.

Synchronicity in the baobab *δ*^13^C records from southwest Madagascar demonstrate regional variability with a succession of wet and dry cycles on the scale of decades to centuries (Fig 4). The rainfall in the region was suggested to be variable with drought events recorded in Southwest Madagascar prior 1000 CE based on stalagmite records [75] along with a hiatus in speleothems and bone isotope records [79,80]. A drying trend was also recorded in the isotope tree records since 1300 CE. Both records, baobab δ^13^C and stalagmite δ¹⁸O show similar trends and patterns demonstrating variability of rainfall over time despite a weak negative Pearson correlation between the two variables (See Table 4). This non correlation could be explained by the uncertainties associated with the two age models as for instance the Asafora chronology includes multiple age reversals and conventional 14C uncertainties that are consistently >100 years. Before 1700 CE, both records show different trends with discrepancies around mid-17^th^ century where the tree record show drier conditions while the stalagmite show wetter conditions. Post 1700 CE similarities between the stalagmite δ^18^O and δ^13^C tree records are recorded with wet condition around 1700–1800 CE, dry condition 1800–1900 CE and a wetter condition again between 1900–2000 CE. Very recently (post-1950 CE), both records reflected a severe drought (Fig 7).

The composite baobab record shows increasing *δ*^13^C values indicating marginal drying at the locations of the trees over the past 700 years, corresponding with previous findings suggesting increasing aridity since 1000 CE, e.g., [30,76,81,82]. Pollen records of the last millennia show a synchronous desiccation from various regions in Madagascar including the southwest [30,79,81] which agrees with reduced length of wet periods in the tree records compared to regional records.

### 4.2. Synoptic drivers of rainfall in southwest Madagascar

When compared with other baobab *δ*^13^C records from South Africa [44,45], the rainfall patterns for southwest Madagascar and the South African summer rainfall zone are in phase for most of the last 700 years. Wet (dry) periods on the multi-decadal to centennial temporal scale in the South African Pafuri and Mapungubwe records (22 °S) corresponded to similar wet (dry) periods from southwest Madagascar. This suggests that southwest Madagascar rainfall responds to similar drivers of the summer rainfall zone in southern Africa, including Agulhas Current sea-surface temperature variations regulated by the East-West displacement of the TTTs [45] in addition to the regulatory effect of the island’s mountains [14,15]. However, further investigation of the various drivers of rainfall is required to provide more understanding of local and regional temporal changes.

### 4.2.1. ITCZ and SAM modulated rainfall during Early Little Ice Age between 1370 and 1500

The composite baobab record from southwest Madagascar shows a wet period between approximately 1350–1500 CE (Fig 4) which coincides with the second phase 1495–1833 CE of a wet-neutral-wet cycle recorded in northwest Madagascar [10] and is also consistent with the end of a dry interval recorded in lake sediment at Ranobe [83]. The movement of the ITCZ has a significant impact on rainfall variability in Madagascar [84–87]. A southward shift was recorded at the beginning of the Little Ice Age around 1300 CE [77,78,88,89] and this may have resulted in increased rainfall for southwestern Madagascar. Wetter conditions are also evident in several East African sediment records during this period, including Lake Chilwa [90], Lake Malawi [91], Lake Massoko [92], Lake Tanganyika [93], Lake Victoria [94], Lake Naivasha [95–97] suggesting a common driver of rainfall, most likely the southwards movement of the ITCZ.

The wetter conditions from 1370–1500 CE in southwestern Madagascar occur when the Southern Annular Mode (SAM) was at its most extreme negative phase in the fifteenth century [70], Fig 8A. Negative SAM indices imply an expansion of the westerly circumpolar vortex, which has significant impacts on temperature and precipitation over all four Southern Hemisphere continents [98], and particularly over Africa south of 25°S, where an increase in precipitation is associated with the northward migration of the westerlies during the austral winter. The response is most pronounced along the east coast, where it is associated with anomalous easterly winds advecting more moisture off the SWIO [98]. No significant response to SAM has been reported for Madagascar on the basis of instrumental data [98] although heavy rainfall events have been associated to atmospheric circulation displays a Southern Annular Mode-like pattern throughout the hemisphere [99]. The relationship between SAM and rainfall in the baobab record presented here is not consistent with an overall significant positive correlation (r = 0.16, p < 0.001). However, since the 16^th^ century the relationship is weak and non-significant suggesting that the southward contraction of the westerly winds during the positive SAM reduces their influence on the region. When the westerlies migrate southward during a positive SAM phase, they cease to be a driver of rainfall, and decadal to centennial rainfall variability responds to other forcing. The lack of consistent correlation throughout the records also supports recent findings related to the lack of a forced response in SAM variability prior to the 20th century [100].

Our composite record suggests that rainfall responds to subtropical forcing during extreme negative SAM phases as the westerly winds migrate northwards bringing wetter condition to the subtropics including southern Madagascar (Fig 8A), but otherwise it responds to tropical forcing determined by the position of the ITCZ during the austral summers. Evidence of this mechanism operating during glacial periods is derived from marine records and model analysis of the Last Glacial maximum over southern Africa [31,101–104]. The mechanism may explain the rainfall maximum during the 14^th^ and 15^th^ centuries, but further research is needed to elucidate the influence of the SAM on southern African climate during the Little Ice Age.

### 4.2.2. Regional and localised rainfall drivers during the LIA 1600–1750

Wet conditions during the 14^th^ and 15^th^ centuries are followed by extremely dry conditions 1600–1750 CE in southwest Madagascar. This period is characterised by a positive phase of the SAM that commences at 1500 CE [70] implying reduced effect of the westerlies. It is also a period of reduced sunspot activity known as the Maunder Minimum (1645–1715) [10,105] with decreased global temperatures at the maximum of the Little Ice Age (LIA, [96]). It has been speculated that there was a southward migration of the ITCZ during the maximum of the LIA [15,16,39,106]. Our results show drier conditions during this period starting around 1600 CE and has also been experienced in northwest of Madagascar around 1700 CE [10]. Dry periods were also experienced across the African continents including East Africa [97,106], and the southern African summer rainfall area [ 45,107–111]. Pollen evidence from the Lake Longiza in southwest Madagascar suggested an increase in grass and decrease in trees and shrubs such as Arecaceae, *Pandanus*, and *Acacia* potentially associated with the drying in the region combined with human activities [112–113]. Lake Tsizavatsy, from the same region, showed a hiatus in its sediment deposition between 1400–1900 CE [76] suggesting the drying of the lake during this period, which is consistent with the tree records. Despite this evidence of drought, decoupled speleothem δ^13^C and δ^18^O records from Anjohibe within the past millennium [114] are inconsistent with climate-driven vegetation changes during recent centuries. The evidence of dry conditions from records across southern Africa does not support southward migration of the ITCZ during this period and if the migration occurred, there might have been other drivers that inhibited its effect leading to a decrease in rainfall during this period.

The Pacific Decadal Oscillation (PDO) is linked to ENSO in relation to drought patterns across Africa through teleconnections that influence the longitudinal position of climate systems. Within the limitations of decadal resolution for comparison, the (PDO) reconstruction shows that during the LIA negative PDO anomalies are associated with decreases in rainfall particularly between 1600–1750 CE (r = −0.30, p < 0.001), whereas in the record before and after this period, they are associated with increases in rainfall (r = 0.18, p = 0.004 and r = 0.18, p = 0.001 respectively) (Fig 8C). Despite the change in the sign of the correlation, this evidence suggests that climate forcing is dominated by tropical forcing [35,71,115]. Changes in the TTTs position which tend to propagate eastward, from southern Africa to the Mozambique Channel and southern Madagascar is known to have a strong influence on intra-seasonal and even interannual rainfall variability in the region [109]. This has particularly been suggested to be dictated by the migration of the ITCZ [24,25] and to increase during La Nina conditions [116,117,118]. Moreover, its persistence was suggested to be maintained by variation of SST anomalies over the Agulhas Current [117,119]. Comparison of the baobab *δ*^13^C records from 1660–1994 CE with the 300-year Agulhas Current Sea surface temperature (SST) record from Ifaty in southwest Madagascar [69] shows a significant negative correlation (r = −0.22, p < 0.001). Positive (negative) SST corresponds with higher (lower) rainfall in the baobab record (Fig 8B) at the decadal level. The coolest oceanic temperatures in the coral record, with anomalies of −0.3 – −0.5 °C between 1675–1760 CE, correspond to the driest period in southwest Madagascar occurring between 1600–1800 CE. Similar patterns of rainfall were noted in the summer rainfall area of the adjacent African mainland [44]. Variation in SST in the western Indian Ocean are determinant in the IOD with a suggested negative relationship with eastern African rainfall responses [115,116].

### 4.2.3. Mixed effect of changes in ITCZ position, human land-use and climate change from 1750–2013 CE

Around 1750 CE until early 1800 CE, at the end of the LIA, there is a relatively wet period recorded in the baobab *δ*^13^C data. A relatively dry period after 1860 CE is similar to conditions experienced over the summer rainfall zone in southern Africa. These periods coincided with more extreme ENSO warm phases [28] with the warmest period in the Agulhas SST record between 1880 CE and 1900 CE and a northward migration of the ITCZ [40,69,120].

The comparison of the composite baobab *δ*^13^C record with the Dipole Mode Index (DMI) that were used as an index of the IOD (1870–2013) shows very low to no correlation (Table 4, Fig 8D) but with a noticeable positive but not significant correlation since 1980 CE (r = 0.27, p = 0.08). The effect that the IOD has on equatorial climate forcing is similar to the ENSO or PDO effects as it is driven by an equatorial SST differential across the Indian Ocean while ENSO is driven by a gradient in the Pacific Ocean. The effect in the subtropical region of southwestern Madagascar appears to be dominated by the Pacific Ocean influences on global climate (ENSO/PDO) which is influential in the latitudinal position of the TTTs system.

Around 1950 CE, conditions in southwest Madagascar are as wet as at any time in the record. This corresponds to a positive SST anomaly, positive phase of IOD and a negative PDO phase. Despite suggested changes in the impact of ENSO cycles on the SST in the region of the SWIO since 1970 [69], our results show typical PDO/rainfall phasing with more negative PDO corresponding to high rainfall while the inverse is not always true. Records suggest that the IOD intensified following the onset of global warming during the 20^th^ century along with forced response of SAM [70,100,121,122], with increased evidence of human induced climate change [1], evidence of increased river runoff and shifts in human land-use through slash and burn agriculture were recorded in coral records from eastern Madagascar [123]. Pollen records from the region suggest a decrease in the tree component including Arecaceae coinciding with an increase in Poaceae and pioneer taxa such as Asteraceae mostly likely associated with tree cutting associated with agriculture expansion [113]. These suggest that changes in rainfall in the region led to changes in land-use. There is a return to drier conditions around 1980 called “belt of iron” also recorded in historical records peaking at the beginning of the 1990s [6]. This was followed by a trend towards wet conditions in the past 20 years similar the instrumental record [16].

### 4.3. Implications for future climate change risk and adaptation

The last 300 years of the baobab composite record resolves the interacting global, regional and local drivers that have influenced rainfall variability in southwest Madagascar at a decadal scale. The dominant effect of the position of the ITCZ during the austral summer is evident, and this is troubling in the context of forecast climate change. The position and zone of the ITCZ is predicted to narrow with a northward shift over eastern Africa and the Indian Ocean and a southward shift in the eastern Pacific and Atlantic Oceans by 2100, which would severely reduce rainfall in the region [124]. In terms of climate risk and adaptation, southwest Madagascar will likely experience a drier climate with more frequent and prolonged droughts as already predicted in the IPCC 6^th^ assessment report [1]. Climatic factors are an important driver of economic, environmental and societal decisions [125] and would be crucial for the future of these dry areas. Indeed, the population is dependent on rainfall as a source of water and for agriculture due to the lack of infrastructure and the limited permanent water ponds [126,127]. The effect of drought and lack of rainfall has already led the Mikea forager communities to diversify their livelihoods with seasonal agriculture to ensure food security [76]. Some adaptations that have been established elsewhere and could be conducted in the region include the introduction of new drought resistant crops, e.g., [128,129]. Multiple species livestock herding with cattle and goat pastoralism [130,131] and livelihood diversification including work that is not farming (e.g., crafts and services for local markets) were suggested to be a major adaptive strategy under drying conditions in a short and long term and buffer livelihoods in the face of environmental change, e.g., [132,133]. Further understanding of the effect of future climate on these populations and their surrounding environment is critical in planning strategies of adaptation in terms of livelihoods but also water provision also in the coming years.

## 5. Conclusion

Baobab δ^13^C data from southwest Madagascar are a proxy for changing rainfall over the last 700 years. The inferred wettest period was between 1370–1500 CE while the driest period occurred between 1600–1750 CE. High centennial variability was recorded with a decreasing rainfall trend and reducing duration of wet periods over time. The comparison of the records with existing records of rainfall drivers at local, regional, and global scales shows that the baobab rainfall proxy record is not dominated by the influence of any single forcing over the entire record. The westerlies may play a role during extreme negative phases of the SAM, while latitudinal shifts of the ITCZ are the dominant low frequency driver of rainfall. At a more local level, the role of SST seems to dominate variability possibly through longitudinal influences on the position of the TTTs system which is also influenced by the PDO/ENSO system. Localised climate forcing in relation to the Southwest Madagascar Coastal Current (SMACC) within the greater Agulhas Current system has been suggested [134]. What emerges from comparisons with other rainfall proxy records on the island of Madagascar, and from the adjacent African mainland, is that the temporal trends are not consistent, probably reflecting the contrasting dominance of different drivers in different regions. A rainfall dipole exists between southern Africa and Madagascar [11,44] with increases in precipitation over southern Africa extending from Mozambique to Angola coincident with a decrease in rainfall most of Madagascar [11] but not in the southwest region. The mountains that extend from the north to the south of Madagascar (>1500 m elevation) reduce the direct transport of moisture from the Indian Ocean toward southern Africa [11] and southwest Madagascar. The Agulhas current SST forcing of Madagascar rainfall is opposite to that in southern African where negative SST anomalies were associated with wetter conditions over the southern African interior [44]. These contradictions suggest that the variation in rainfall is not a simple intensification or weakening of the existing climate patterns, but rather a response in the synoptic systems to tropical (ENSO/PDO), extratropical (SAM), and localised (SST) forcing. Why some forcing appears to dominate at certain periods and not at others is unclear, and the evidence presented here suggests that synergistic effects might be explored in global climate models.

The potential effect of climate change and land-use change were also recorded at the near present period, as well as possible effects of SAM if there is a northward migration of westerlies similar to what happened around 1300 CE. The data generated here provide the opportunity to unravel the relative importance and interaction between global, regional, and local drivers across the southern and eastern African region. These findings are crucial in the simulations of rainfall projections to help evaluate the impacts and trends of migration of the westerlies and anthropogenic induced climate change on the African continents that are not fully understood. For southwest Madagascar with an expected drier climate and increasing occurrence of severe drought conditions predicted for the near future, understanding the risks, and establishing adaptation strategies particularly in terms of livelihood could avoid disastrous famine.

## Supporting information

S1 Table AMS Radiocarbon dates for the 4 cores covering in total 52 dates. (DOCX)

S2 Checklist Research Permit obtained from the ministry of environment and sustainable development for sample collection. (DOCX)
