# Supplementary material for: Baobab isotope records and rainfall forcing in Southwest Madagascar over the last 700 years
Source: PLoS One. 2026 Mar 10;21(3):e0331274. doi: 10.1371/journal.pone.0331274 (PMC12974867; doi:10.1371/journal.pone.0331274)
Supplement: S1 Table — (DOCX) [file pone.0331274.s001.docx]

Supplementary information I (SI1)1: Radiocarbon dates of each tree replicate from the four trees collected in southwest Madagascar

| Sample ID | **Core/ring number** | **Date (uncalibrated years BP)** | **AD Calibrated date 1sig, **2sig** | **Assigned year** | **Lab ID** |
| --- | --- | --- | --- | --- | --- |
| DFL | 62 | 133.±0.65pmc | 1963 | 1928 | IT-C-848 |
|  | 121 | 1860.1±43.7 | 1743 | 1840 | IT-C-851 |
|  | 124 | 499±73.9 | 1627** | 1835 | IT-C-365 |
|  | 127 | 95.1±33.8 | 1837 | 1831 | Not noted |
|  | 151 | 80.4±31 | 180968** | 1795 | IT-C-539 |
|  | 199 | 127.3±38.2 | 1721 | 1723 | IT-C-1214 |
|  | 250 | 253.6±30.2 | 1649 | 1647 | IT-C-1213 |
|  | 300 | 376.7±31.4 | 1542 | 1560 | IT-C-542 |
|  | 374 | 546.2±40.3 | 1408 | 1422 | IT-C-850 |
|  | 377 | 518.9±50.8 | 1409 | 1416 | IT-C-878 |
|  | 432 | 668±43.4 | 1301 | 1314 | IT-C-145 |
|  | 433 | 650.4±31.3 | 1318 | 1312 | IT-C-536 |
| DFS | 40 | 128.9±0.48pmc | 1979 | 1977 | IT-C-529 |
|  | 111 | 101.1±0.51pmc | 1955** | 1908 | IT-C-846 |
|  | 116 | 140.2±62.9 | 1947** | 1904 | IT-C-856 |
|  | 201 | 161.6±28.8 | 1807 | 1805 | IT-C-1216 |
|  | 205 | 349.8±76.2 | 1803** | 1800 | IT-C-171 |
|  | 251 | 57.8±40.4 | 1724 | 1745 | IT-C-847 |
|  | 255 | 207.7±31.8 | 1726 | 1740 | IT-C-858 |
|  | 275 | 118.6±29.3 | 1710** | 1716 | IT-C-1215 |
|  | 315 | 100.5±0.76pmc | 1698* | 1668 | IT-C-624 |
|  | 338 | 226±30.8 | 1647* | 1640 | IT-C-541 |
|  | 403 | 461.2±45.5 | 1437 | 1465 | IT-C-860 |
|  | 445 | 793±133 | 1324 | 1237 | IT-C-173 |

| Sample ID | **Core/ring number** | **Date (uncalibrated years BP)** | **AD Calibrated date 1sig, **2sig** | **Assigned year** | **Lab ID** |
| --- | --- | --- | --- | --- | --- |
| GTR | 50 | 106.4±0.41pmc | 2003* | 1992 | IT-C-544 |
|  | 148 | 148.4±0.52pmc | 1963 | 1920 | IT-C-573 |
|  | 151 | 153.9±1.61pmc | 1964 | 1918 | IT-C-367 |
|  | 183 | 211.3±42.8 | 1875 | 1894 | IT-C-1050 |
|  | 188 | 99.83±0.53pmc | 1878 | 1891 | IT-C-1072 |
|  | 219 | 101.3±0.62pmc | 1955 | 1868 | IT-C-788 |
|  | 250 | 122.7±28.3 | 1850 | 1845 | IT-C-1222 |
|  | 275 | 177.5±34.7 | 1835 | 1827 | IT-C-1221 |
|  | 295 | 844.6±31.5 | 1270 | 1812 | IT-C-575 |
|  | 299 | 112.1±96.9 | 1805 | 1809 | IT-C-361 |
|  | 359 | 237.6±31.7 | 1759 | 1765 | IT-C-540 |
|  | 403 | 526.6±54.9 | 1454 | 1447 | IT-C-845 |
|  | 447 | 903.1±192 | 1379 | 1347 | IT-C-668 |
| TSP | 26 | 137±0.44pmc | 1976 | 2004 | IT-C-1273 |
|  | 77 | 103.4±0.37pmc | 1958 | 1981 | IT-C-1223 |
|  | 126 | 100.2±0.4pmc | 1957 | 1959 | IT-C-1219 |
|  | 159 | 104.6±0.61pmc | 1956 | 1944 | IT-C-1217 |
|  | 226 | 102.8±0.37pmc | 1955 | 1914 | IT-C-557 |
|  | 276 | 82.3±30.8 | 1890 | 1892 | IT-C-1218 |
|  | 291 | 34.3±28 | 1884* | 1881 | IT-C-574 |
|  | 298 | 125.8±30.9 | 1637* | 1875 | IT-C-374 |
|  | 402 | 373.4±40.1 | 1784 | 1787 | IT-C-849 |
|  | 405 | 287.5±28.3 | 1712 | 1785 | IT-C-881 |
|  | 500 | 79.7±31.3 | 1712 | 1704 | IT-C-558 |
|  | 512 | 186.8±44.2 | 1671 | 1694 | IT-C-172 |
|  | 601 | 293.3±41 | 1624 | 1617 | IT-C-556 |
|  | 675 | 717.2±74.9 | 1392 | 1394 | IT-C-1051 |
|  | 705 | 253.7±31.1 | 1633 | 1303 | IT-C-1058 |

** indicate dates from 2sigma-calibrated error
