# Supplementary material for: Baobab isotope records and rainfall forcing in Southwest Madagascar over the last 700 years
Source: PLoS One. 2026 Mar 10;21(3):e0331274. doi: 10.1371/journal.pone.0331274 (PMC12974867; doi:10.1371/journal.pone.0331274)
Supplement: Supplementary file 2 [file pone.0331274.s002.docx]

REPOBLIKAN'I MADAGASIKARA
Fitiavana-Tanindrazana–Fandrosoana

SECRETARIAT GENERAL

DIRECTION GENERALE DES FORETS

DIRECTION DES AIRES PROTEGEES TERRESTRES

AUTORISATION DE :
x- RECHERCHE
- ETUDE

N° 211 /15/ MEEMF /SG/DGF/DAPT/ SCBT

NOM RAZANATSOA
PRENOMS Estelle
ADRESSE B.P :175 Antananarivo
FONCTION Chercheur-Doctorante
ACCOMPAGNE DE : Randriamalaza Tafitaniaina, Lindsey Gillson, Grant Hall, un étudiant, un représentant du CAFF/CORE.

ORGANISME TUTELLE : ESSA/ Département des Eaux et Forêts

EST AUTORISE(E) A FAIRE DES RECHERCHES / ETUDES DANS :
Le réseau des Parc Nationaux : Kirindy Mitea, Tsimanampetsotsa

MENTION SPECIALE EVENTUELLE:
Etude des impacts de la variation climatique sur les écosystèmes du Sud Ouest de Madagascar.
Comprendre le rôle de la précipitation ainsi que des activités humaines sur les écosystèmes au cours des 2000 dernières années.
Collecte d'échantillons de carottes sédimentaires, où la préservation des pollens est bonne
Collecte d'échantillons de surface pour obtenir de pollens moderne de la végétation
Collecte d'échantillons de carottes sur le tronc de baobab pour analyse des cernes
Collecte d'échantillons botanique pour herbier d'au maximum cinq spécimens par espèce.

DUREE : Six (06) mois.

N.B : L'ESSA-Département des Eaux et Forêts doit remettre à la Direction des Aires Protégées Terrestres, en quatre (04) exemplaires EN FRANÇAIS, le rapport préliminaire à la fin de sa mission et le rapport final avec les résultats des recherches au plus tard deux ans après la mission. Rapports en versions papier et électronique.
Le bénéficiaire de la présente autorisation doit :
- faire viser la présente par la Direction Régionale de l'Environnement, de l'Ecologie, de la Mer et des Forêts Atsimo Andrefana et/ou CEEMF concernés avant toute descente sur terrain.
- prendre le ticket d'entrée auprès de MNP (Madagascar National Parks) dans le cas où la recherche s'effectue dans les Aires Protégées gérées par celui-ci conformément à la note **n° 394-10/MEF/SG/DGF/DVRN/SGFF du 18 Mai 2010.**

AMPLIATIONS :
- CAFF/CORE
- DCF
- DREEMF : Aand
- CEEMF concernée
- MNP
- A.P concernées
- Communes concernées
- **« Pour contrôle et suivi »**

- ESSA – Département des Eaux et Forêts
« Pour le rapport

Antananarivo, le 27 AOUT 2015

LE DIRECTEUR DES
AIRES PROTEGEES TERRESTRES

RAMIANDRASIVO [illegible] Herinirina
Ingénieur [illegible]
